# Long-term efficacy of etanercept in ADA2 deficiency

**DOI:** 10.1186/1546-0096-12-S1-P72

**Published:** 2014-09-17

**Authors:** Roberta Caorsi, Alessia Omenetti, Paolo Picco, Antonella Buoncompagni, Francesca Minoia, Silvia Federici, Martina Finetti, Alberto Martini, Ivona Aksentijevich, Marco Gattorno

**Affiliations:** 1 2nd Division Of Pediatrics, ISTITUTO GASLINI, GENOA, Genova, Italy; 2 National Human Genome Research Institute, Bethesda, USA

## Introduction

Mutations of ADA2 have been recently reported as causative of an inflammatory condition characterized by polyarteritis, cerebral stroke and immunodeficiency; the response to immunosuppressors and biologic drugs is not univocal.

## Objectives

To describe a series of patients with DADA2, focusing on the response to treatment.

## Methods

We retrospectively collected the clinical course and response to treatment of three paediatric patients with DADA2.

## Results

A boy born from non-consanguineous parents at the age of 2 years presented with livedo reticularis associated to elevation of acute phase reactants and recurrent episodes of arthralgia, fever and hypertension responsive to prednisone. At the age of 6 years he presented a haemorrhagic stroke of the left temporal lobe. A skin biopsy was consistent with a diagnosis of polyarteritis. The patient presented good response to thalidomide. Low immunoglobulins levels were then detected,.Two years later, treatment was discontinued with a prompt recurrence of the symptoms, requiring high dose steroids. Etanercept was then started (25 mg/week) with normalisation of both clinical picture and laboratory parameters. The levels of plasmatic immunoglobulins slowly normalized. Even the hypertension improved. The molecular analysis of the *CECR1* gene revealed the presence of two heterozygous mutations (R312X and E328D). After five years of treatment the patient is in wellbeing. The younger brother of the above patient at the age of 9 months presented with livedo reticularis, hypertension, reduction of plasmatic immunoglobulin levels and elevation of acute phase reactants, responsive to short steroidal course. At the age of one year the child showed intestinal ischemia, secondary to vasculitis, consistent with PAN. After few months the patient presented a stroke with hemiplegia. Treatment with thalidomide controlled the clinical picture for two years, but was then discontinued due to a reduced nerve conduction velocity. Soon after discontinuation livedo reticularis and elevation of acute phase reactants were observed. Treatment with etanercept (12.5 mg/week) was therefore started with prompt normalisation of acute phase reactants; hypertension and the levels of plasmatic immunoglobulins slowly normalized. Within three years from the beginning of the treatment, the patient is in wellbeing. CERC1 analysis revealed the same mutations of the brother. A third non-related child born from consanguineous parents at the age of 6 month presented with facial hemiparesis. Persistent elevation of acute phase reactants were also observed. At the age of 1 year the patient presented an episode of myocarditis treated with diuretics and antibiotics. At the age of 2 livedo reticularis occurred. In the following years he presented a persistent elevation of acute phase reactants and developed two episodes of ischemic stroke High doses of steroids were were able to control the clinical picture with elevation of acute phase reactants at tapering. Treatment with etanercept was therefore stared (25 mg/weekly) with prompt normalization of acute phase reactants and persistent wellbeing after 8 month of treatment. Analysis of CERC1 gene revealed the T360A homozygous mutation.

## Conclusion

This series of three cases enlightens the long-term efficacy of etanercept in ADA2 deficiency.

## Disclosure of interest

None declared.

